# A technique combining “U” shape suture and shared tunneling to treat the posterior cruciate ligament rupture and posterior root tears of the medial meniscus

**DOI:** 10.1186/s13018-018-0973-0

**Published:** 2018-10-22

**Authors:** Xing-He Xue, Jian Lin, Wei-Hui Qi, Xiao-Yun Pan

**Affiliations:** 1 Department of Orthopaedics, The Second Affiliated Hospital and Yuying Children’s Hospital of Wenzhou Medical University, 109 Xueyuan Xi Road, Wenzhou, 325000 People’s Republic of China; 2 Zhejiang Provincial Key Laboratory of Orthopaedics, 109 Xueyuan Xi Road, Wenzhou, 325000 People’s Republic of China; 3 The Second School of Medicine, Wenzhou Medical University, 109 Xueyuan Xi Road, Wenzhou, 325000 People’s Republic of China

**Keywords:** Arthroscopy, Posterior root of the medial meniscus, Posterior cruciate ligament, Bone tunnel

## Abstract

**Background:**

The standard treatment of the posterior cruciate ligament (PCL) rupture accompanied with the posterior root of medial meniscus (PRMM) tears is controversial. Our research describes a minimally invasive technique for the PCL rupture accompanied with the PRMM tears.

**Methods:**

We described a “U” shape suture and shared tunneling technique to treat the PCL rupture accompanied with PRMM tears. Three patients (ages 28, 42, and 59 years old) who underwent this surgery have been followed up for more than 1 year at most. The MRI was done, and the hospital for special surgery (HSS) score was adopted to evaluate the clinical effect. Firstly, we built both femoral and tibial bone tunnels for the PCL reconstruction. Secondly, we used the suture hook to pass the suture line through the PRMM. Thirdly, we passed the prepared tendon through the bone tunnel and fixed the prepared tendon by an endobutton plate and an interference screw (Smith & Nephew) respectively on the side of the femur and tibia. At last, we used an endobutton plate (Smith & Nephew) outside the tibial bone tunnel to fix the PRMM.

**Results:**

These three patients did not show any complications. At 1 year after the operation, we found good knee stability, negative posterior drawer test, and normal range of motion compared with the contralateral knee joint. The MRI also showed a good union of the PRMM and PCL. The hospital for special surgery (HSS) score was 90 points.

**Conclusions:**

With an ideal therapeutic effect, this technique is worthy to be promoted for patients with the PCL rupture and PRMM tears.

**Electronic supplementary material:**

The online version of this article (10.1186/s13018-018-0973-0) contains supplementary material, which is available to authorized users.

## Background

The posterior cruciate ligament (PCL) rupture is one of the most common knee joint diseases which plays an important role in maintaining the posterior and rotatory stability of the tibia [1–3]. The meniscus is crucial to load bearing, load transmission, and shock absorption of the knee joint. The buffer action of the meniscus helps protect the knee joint from acute or chronic injury [4]. The PCL rupture may be accompanied with meniscus tears, including the posterior root of medial meniscus (PRMM) tears, due to its relation with the anatomical position. The PRMM is located 9.6 mm posterior and 0.7 mm lateral from the medial tibial eminence. The mean distance between the central point of PRMM and the edge of PCL is about 8.2 mm [5, 6]. The MRI study finds that the most common attachment of the PRMM is the posterior area of tibial intercondylar eminence, but 14.7% of the patients turn out to be both with posterior area of tibial intercondylar eminence and the PCL [7, 8]. This specific anatomical position results in the concomitant occurrence of the PCL rupture accompanied with PRMM tears.

The PRMM tears will lead to loss of hoop tension, loss of load sharing ability, and unacceptable peak pressures [1]. As a result, it is necessary to fix the PRMM area apart from reconstructing the PCL. The standard surgical technique of the PRMM tears is still controversial which mostly focuses on the anchor technique and bone tunnel technique [6–8]. However, there are no surgical descriptions of the PCL rupture accompanied with PRMM tears.

We describe a “U” shape suture and shared tunneling technique to treat the PCL rupture accompanied with the PRMM tears and prove its advantages retrospectively.

## Methods

### Patient preparation

**Fig. 1:**
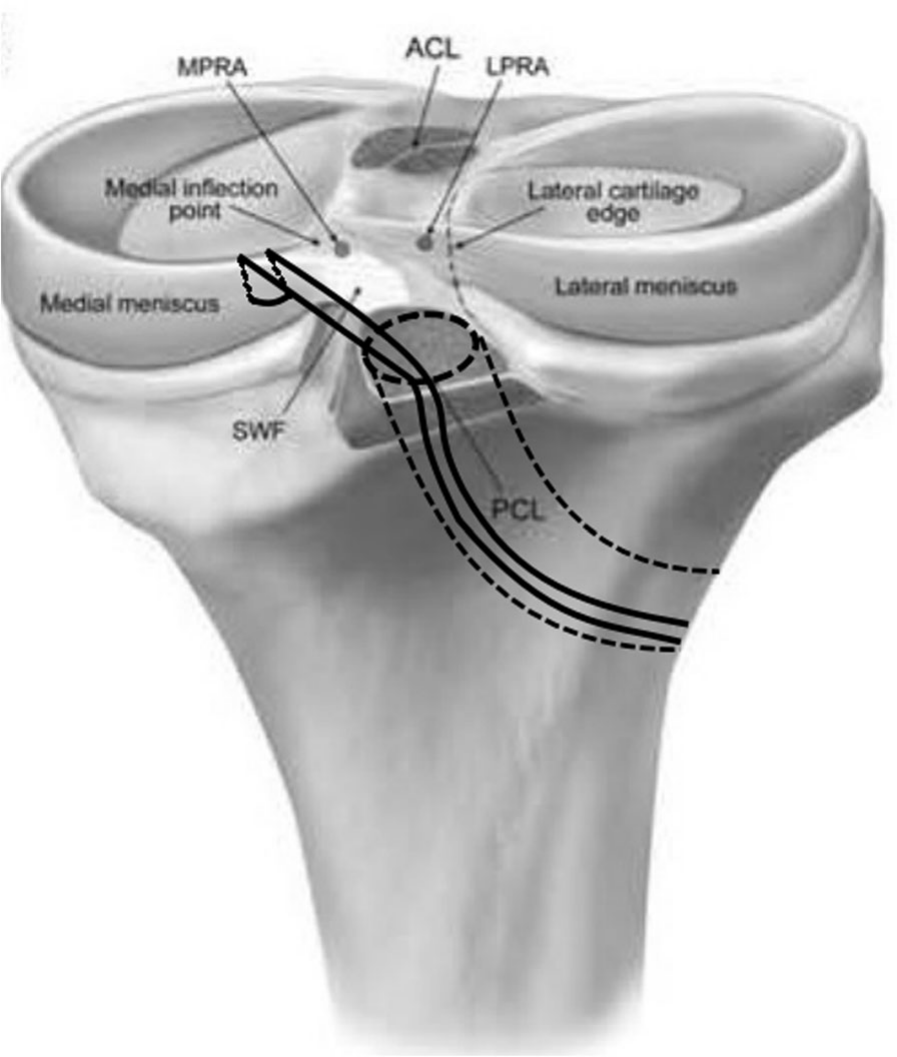
The sketch of the “U” shape suture and shared tunneling technique described in our article

This surgical technique was performed in three patients from 2015 to 2017. All the patients were male of ages 28, 42, and 59 years old. Only patients diagnosed with PCL rupture accompanied with PRMM tears were included. The follow-up was 1 year, and the hospital for special surgery (HSS) score was adopted to evaluate the outcome at 1 year. The patient was positioned supine, and the combined spinal-epidural anesthesia was done. Then, we used the standard anteromedial and anterolateral portals of arthroscopic operation to confirm our preoperative diagnosis. All the patients were diagnosed with PCL rupture accompanied with PRMM tears. We combined “U” shape suture and shared tunneling technique to cure this disease (Fig. 1).

### Description of the surgical technique

Firstly, we performed the PCL reconstruction in a routine manner. We used tibial localizer and femoral localizer to drill tibial bone tunnel and femoral bone tunnel. The diameter of the bone tunnel was 8 or 9 mm which were dependent upon the graft size and diameter. After we drilled both femoral bone tunnel and tibial bone tunnel for the PCL reconstruction, we started the arthroscopic meniscoplasty for the PRMM tears.

**Fig. 2:**
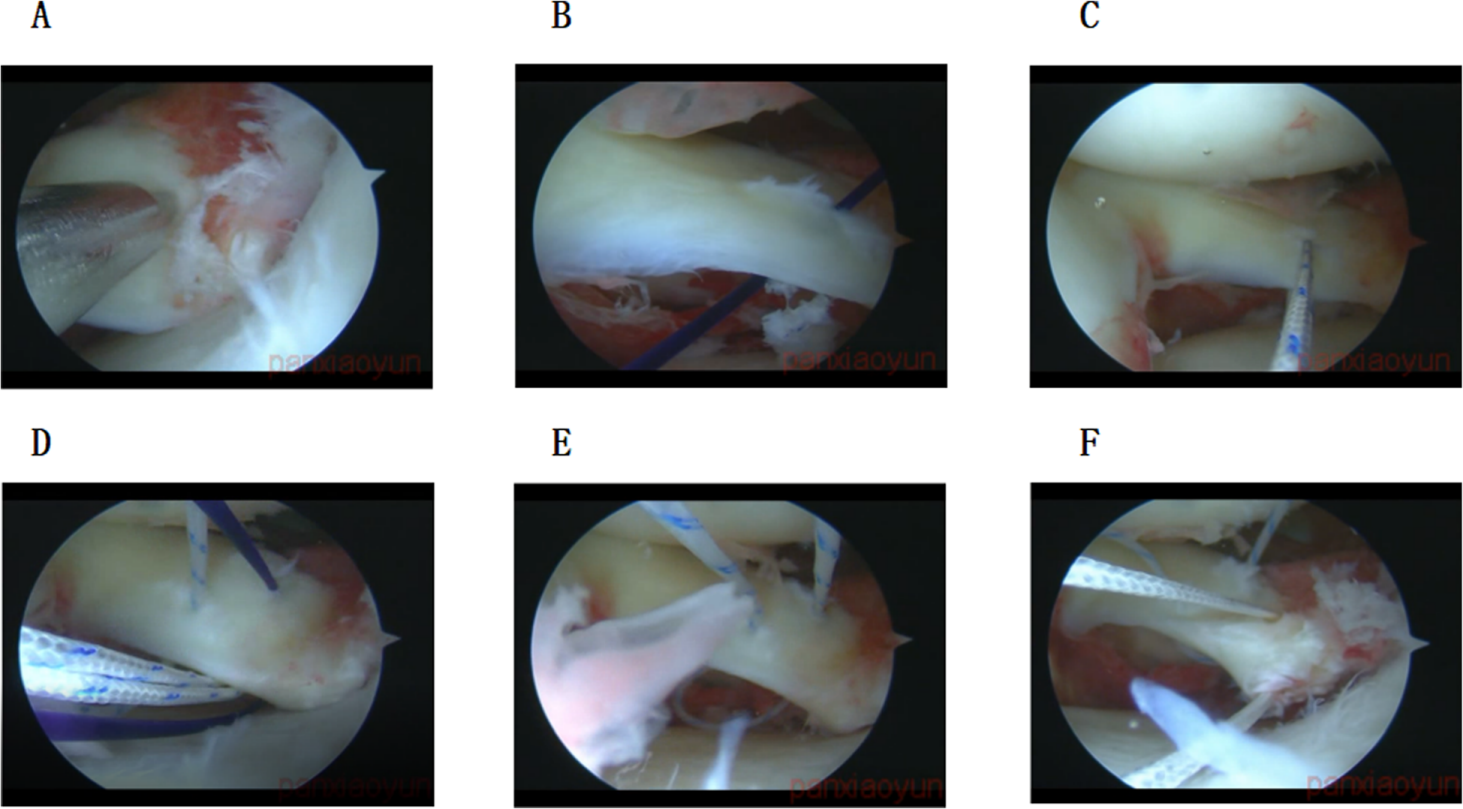
The description of the “U” shape suture. **a**–**e** We used the suture hook, PDS suture line, and Smith & Nephew suture line to do the “U” shape suture. **f** An extra suture line was used to help the reduction

The suture hook and line grasping device in shoulder arthroscopy were used in arthroscopic meniscoplasty to do “U” shape suture (Additional file 1: Video S1). Firstly, we used the arthroscopic probe to relocate the PRMM in order to evaluate the appropriate suture location. Secondly, we used the suture hook to pass the PDS suture line through the PRMM (Fig. 2a, b). Then, we replaced the PDS suture line to the Smith & Nephew suture line (Fig. 2c). Thirdly, the same line passing technique was used to pass another PDS suture line through the medial meniscus root area near the previous location. The midpoint of two entry points should be as close as possible to the central point of the PRMM. Lastly, we used the line grasping device to grasp both line ends below the meniscus through one portal and replace the PDS suture line with the same Smith & Nephew suture line (Fig. 2d). As a result, both line ends were above the PRMM, and we called it “U” shape suture (Fig. 2e). The two line ends above the PRMM helped to press the PRMM close to the tibial cartilage surface when compared with the situation of the two line ends below the PRMM. This idea is borrowed from the double-row fixation technique in shoulder arthroscopy. Besides, if you found the reduction of the PRMM is not good, you can pass another suture line through the PRMM to help the reduction (Fig. 2f).

**Fig. 3:**
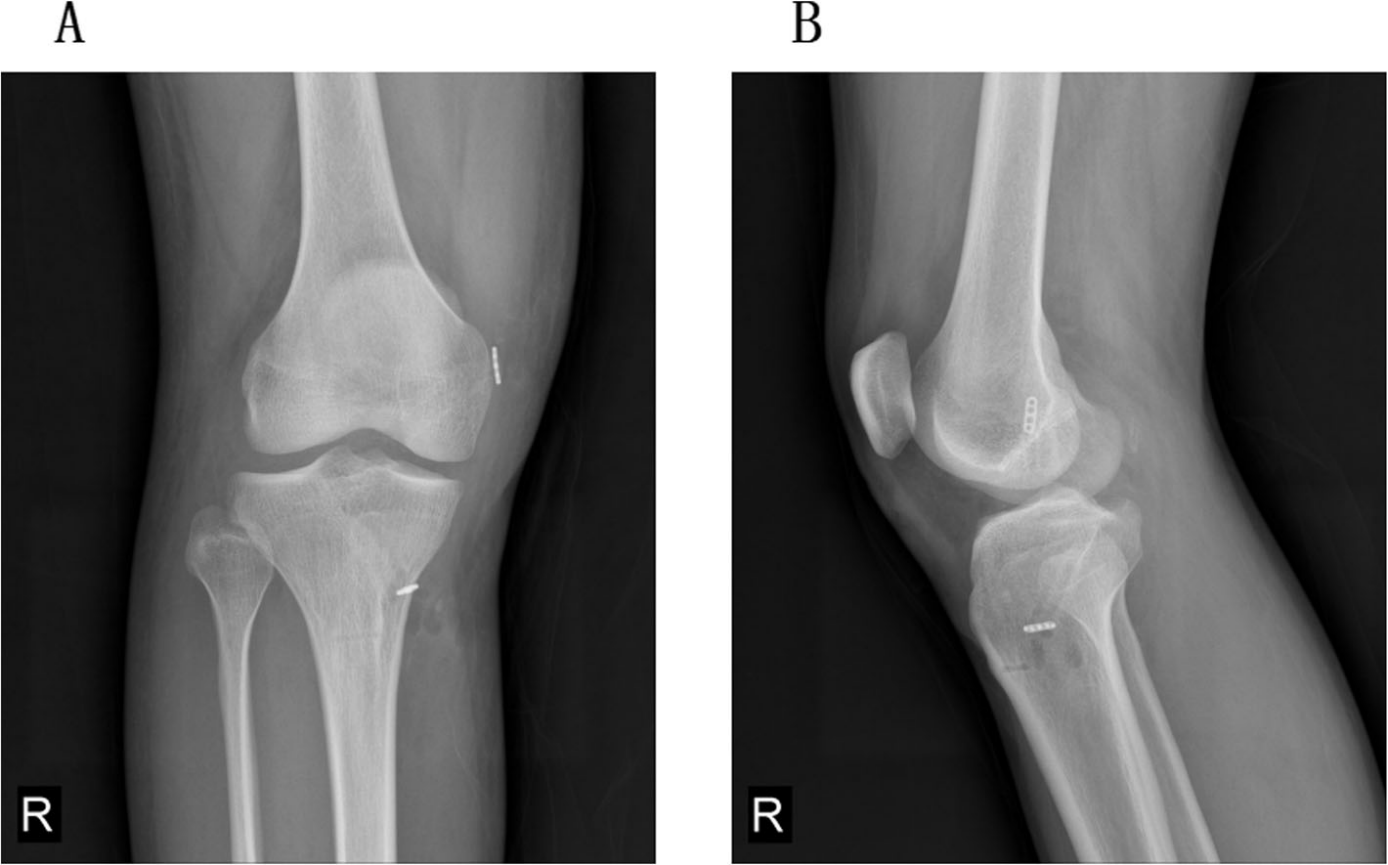
The postoperative X-ray showed an endobutton plate (Smith & Nephew) outside the tibial bone tunnel, which was used to fix the suture line passed through PRMM area. **a** anteroposterior film. **b** lateral film.  PRMM, posterior root of medial meniscus

**Fig. 4:**
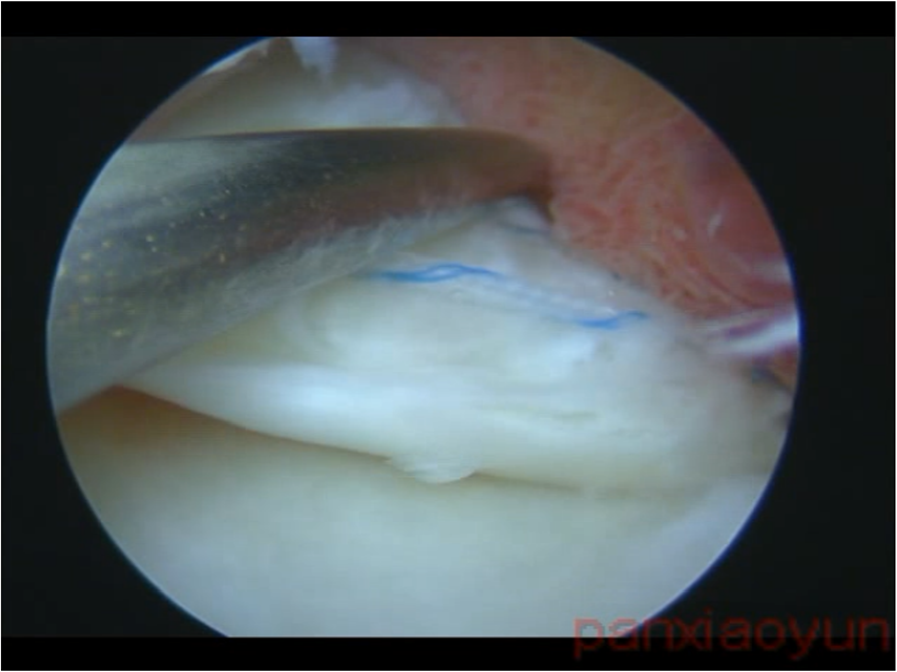
The PRMM turned out to be stable when checked by an arthroscopic probe. PRMM, posterior root of medial meniscus

After the “U” shape suture, we used shared tunneling technique to fix the medial meniscus root area. We firstly passed both ends of the suture line through the tibial bone tunnel (Fig. 1). After which, we passed the prepared allograft patellar tendon through the bone tunnel and fixed the prepared tendon by an endobutton plate and an interference screw (Smith & Nephew) respectively on the side of the femur and tibia. During the entire process, we must maintain a certain tension of the suture line to prevent the loosening of the PRMM. An endobutton plate (Smith & Nephew) outside the tibial bone tunnel was used to fix the PRMM (Fig. 3). At last, we used the probe to evaluate the stability of the meniscus and PCL, and it turned out to be ideal (Fig. 4).

### Rehabilitation

The plaster immobilization and non-weight bearing were adopted in the first 2 weeks after surgery. Then, we started passive motion and partial weight bearing. After 6 weeks, we started active flexion and extension and full weight bearing in straight position.

## Results

**Fig. 5:**
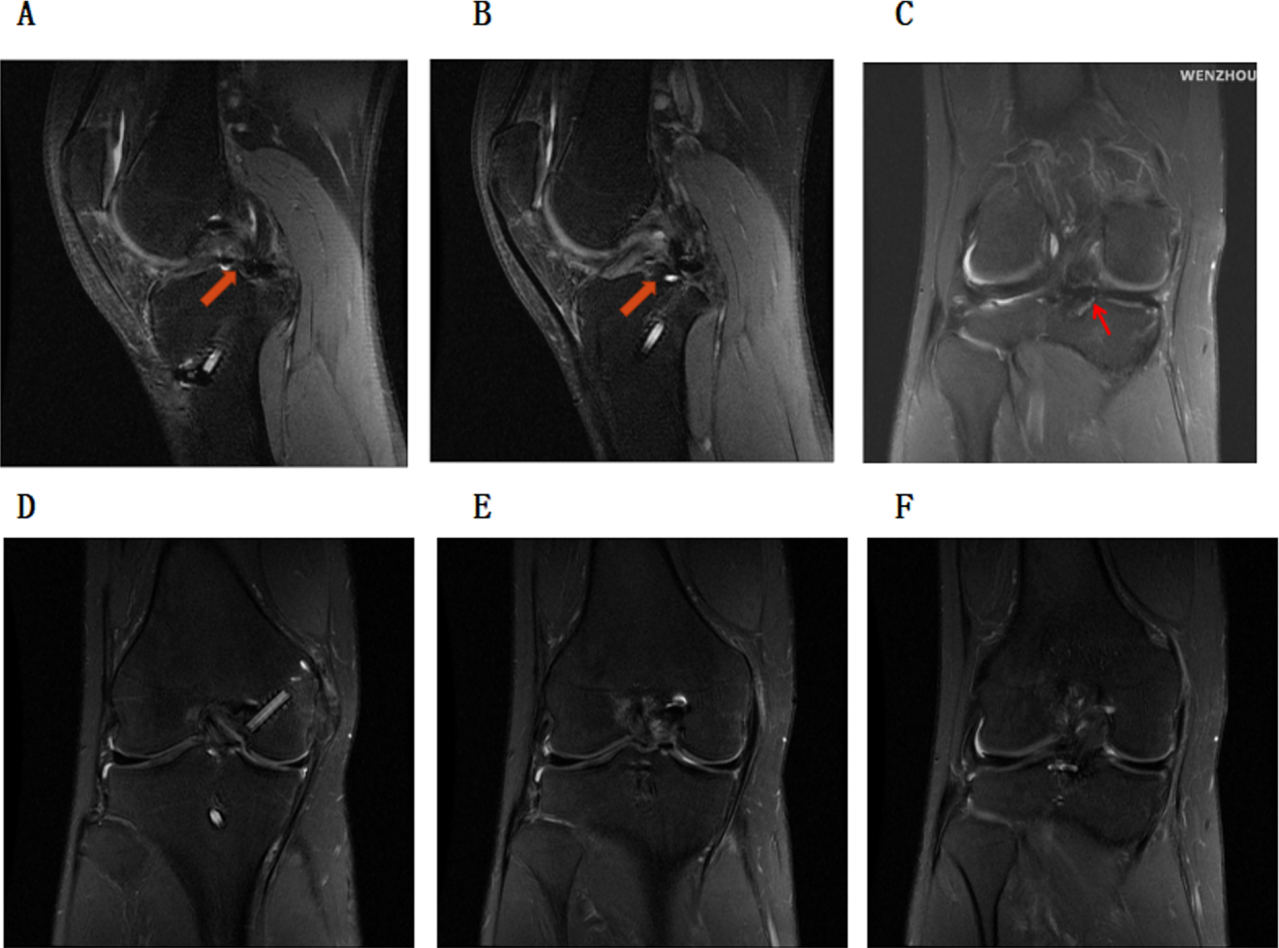
The postoperative MRI at 1 year. **a**–**c** The red arrows show the good union of the PRMM. **d**–**f** The good union of the PCL. PRMM, posterior root of medial meniscus, PCL, posterior cruciate ligament

There were no intraoperative complications, and the operation time was about 1 h. The longest follow-up time was more than 1 year and the shortest was 1 month. No patient had recurrent meniscal tears or PCL recurrent rupture, physical examination, or MRI. During the several days after the operation in the hospital, there were no any complications related to this technique. The 28-year-old patient followed up for 1 year had already returned to previous activity level and did not have any complications. At 1 year after surgery, the postoperative MRI showed perfect healing of the PRMM and PCL (Fig. 5). The posterior drawer test showed a good result. And the hospital for special surgery (HSS) score of this patient at 1 year after surgery was 90 points.

## Discussion

Compared with the previously described surgical techniques, our surgical technique has several advantages: (1) standard arthroscopic portals for PCL reconstruction without additional incision; (2) the “U” shape suture technique increases the contact surface between the PRMM and bone surface, which is in favor of the union; (3) the shared tunneling technique avoids additional bone tunnel which may influence the bone tunnel for PCL reconstruction; (4) stronger fixation. However, there may be some limitations which cannot be avoided. The shared tunneling technique may damage the suture line which passes through the PRMM area. Another potential complication can be that if appropriate tension is not maintained on the suture line while fixing the PCL, then the PRMM would become loose and not fixed appropriately on the floor of its insertion.

The menisci play an important role in protecting the cartilage surfaces of the knee joint from axial loads [9]. Recently, the treatment of the PRMM is not uniform including conservative treatment, meniscectomy, and meniscus repair [10–13]. The conservative treatment did not show any benefit but led to a high rate of knee arthritis. Thirty-one percent of the patients who chose conservative treatment undertook total knee arthroplasty [13]. Allaire et al. found that the repair of the posterior meniscal root including PRMM is in favor of the recovery of the peak contact pressures of the knee joint [14]. The same result was found in the study of Chung et al. which compared meniscus repair with meniscectomy [10]. The good reduction and recovery of the PRMM can reduce the risk of arthritis.

Nowadays, most surgical techniques focus on suture anchor or transtibial bone tunnels, but the best surgical technique is still under discussion. Several biomechanical researches have been performed, but no unified conclusion is found. One prospective clinical study compared the two techniques. After 2 years follow-up, there was not any statistically significant difference between the two techniques in functional improvement or healing rates. But postoperative recovery improved compared with the preoperative state (*p* < 0.05) [6]. The curative effects of both techniques are worthy to be affirmed. However, when the PRMM tears are complicated with the PCL rupture, the transtibial bone tunnel technique seems to be the first choice which is with lower damage and higher convenience. Our first patient who was followed up for 23 months showed good recovery. The postoperative MRI at 1 year showed a good union of the PRMM and the PCL, and the patient could do all the exercises as he used to do preoperatively.

## Conclusions

With an ideal therapeutic effect, this technique is worthy to be promoted for patients with the PCL rupture and PRMM tears.

## Additional file

Additional file 1: The surgical procedure of our technique. (MP4 8779 kb)

## Abbreviations

PCL: Posterior cruciate ligament
PRMM: Posterior root of medial meniscus
